# Crystal structure, spectroscopic characterization and DFT study of two new linear fused-ring chalcones

**DOI:** 10.1107/S2056989018012641

**Published:** 2018-09-14

**Authors:** Dian Alwani Zainuri, Ibrahim Abdul Razak, Suhana Arshad

**Affiliations:** a X-ray Crystallography Unit, School of Physics, Universiti Sains Malaysia, 11800 USM, Penang, Malaysia

**Keywords:** chalcone, crystal structure, DFT, mol­ecular electrostatic potential

## Abstract

The structures of two new anthracenyl chalcones, namely (*E*)-1-(anthracen-9-yl)-3-(4-nitro­phen­yl)prop-2-en-1-one, C_23_H_15_NO_3_, and (*E*)-1-(anthracen-9-yl)-3-(4-iodo­phen­yl)prop-2-en-1-one, C_23_H_15_IO are reported. A structural comparative study between the two chalcones was performed and some effects on the geometrical parameters, such as planarity and dihedral angles, are described. The mol­ecular geometry was determined by single-crystal X-ray diffraction, and density functional theory (DFT) at B3LYP with the 6–311++G(d,p) basis set was applied to optimize the ground-state geometry. In addition, inter­molecular inter­actions responsible for the crystal packing were analysed. The electronic properties, such as excitation energies and HOMO–LUMO energies were calculated by time-dependent density functional theory (TD–DFT) and the results complement the experimental findings. The mol­ecular electrostatic potential (MEP) was also investigated at the same level of theory in order to identify and qu­antify the possible reactive sites.

## Abstract 2

A structural comparative study between two chalcones was undertaken and some effects on geometrical parameters, such as planarity and dihedral angles, are described.

## Chemical context   

The synthesis of new organic mol­ecules and the characterization of their mol­ecular properties are the necessary prerequisites for further research in modern technologies. Conjugated organic chalcone mol­ecules are recognized to be promising materials in the field of opto-electronic applications (Aggarwal *et al.*, 2001 ▸). The materials are characterized by an extremely excited π-conjugated chain with strong electron acceptor–donor pairs at the end (*D*–π–*A*) of the terminal rings (Manjunath *et al.*, 2011 ▸). Chalcone derivatives are an inter­esting type of organic NLO materials that can be tuned to match particular requirements. In these systems, two aromatic rings have to be substituted with suitable electron-donor or acceptor groups to increase the asymmetric charge distribution in either or both the ground state and excited states, giving rise to an enhanced optical non-linearity (Rajesh Kumar *et al.*, 2012 ▸). Meanwhile, the enone moiety acts as the π-conjugated bridge that is responsible for inter­molecular charge transfer between the donor and acceptor substituent groups. The title compounds contain an anthracene fused-ring system (strong electron donor) containing a nitro group or an iodine atom (strong electron acceptor) substituted at the *para* terminal position. Their investigation included characterization using UV–vis spectroscopy and computed studies of HOMO–LUMO energy gaps and mol­ecular electrostatic potential (MEP).
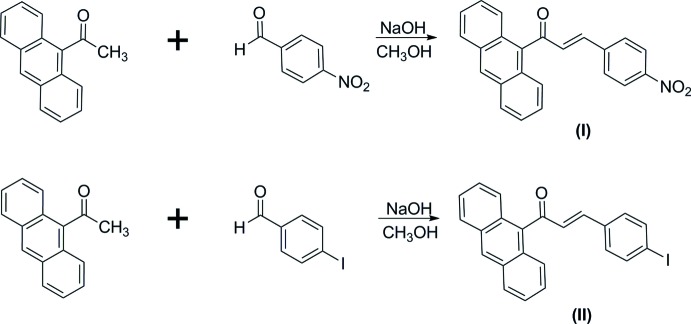


## Structural commentary   

**Figure 1:**
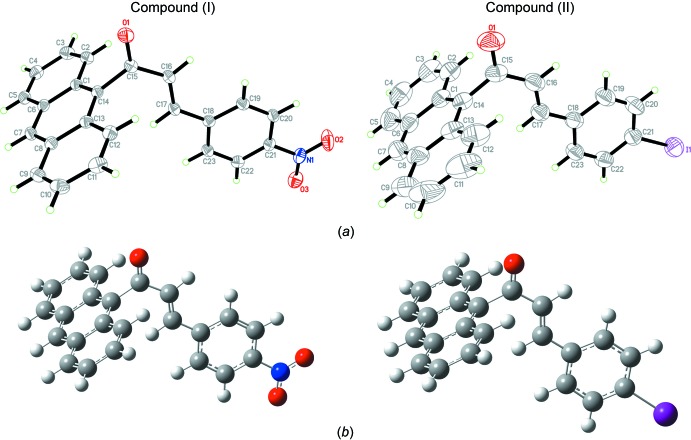
(*a*) The mol­ecular structures of compounds (I) and (II) and (*b*) the optimized structures of (I) and (II) at the DFT/B3LYP 6–311++G(d,p) level.

The mol­ecular structures of the compounds (I) and (II) are shown in Fig. 1 ▸ *a*. All geometrical parameters are within normal ranges and comparable with those in the previously reported structure of anthracenyl chalcones (Zainuri *et al.*, 2018*a* ▸). The optimization of the mol­ecular geometries (Fig. 1 ▸ *b*) leading to energy minima was achieved using DFT [with Becke’s non-local three parameter exchange and the Lee–Yang–Parr correlation function (B3LYP)] with the 6-311++G (d,p) basis set as implemented in *Gaussian09* program package (Frisch *et al.*, 2009 ▸).

The compounds exist in an *s-trans* configuration with respect to the C15=O1 [experimental = 1.2246 (17) and DFT = 1.22 Å in (I); exp = 1.226 (3) and DFT=1.22 Å in (II)] and C16=C17 [exp = 1.335 (2) and DFT = 1.34 Å in (I); exp = 1.336 (4) and DFT= 1.35 Å in (II)] bond lengths within the enone moiety. The mol­ecular structures of both compounds are twisted at the C14—C15 bond with C1—C14—C15—C16 torsion angles of −94.21 (16) and 97.3 (3)° in (I) and (II), respectively. The corresponding DFT values are −91.63° (I) and −85.63° (II). The large twist angles are a result of the bulkiness of the strong-electron-donor anthracene ring system (Zainuri *et al.*, 2018*b* ▸). The enone moieties are found to be essentially planar with respect to the C17=C18 double bond with the C16—C17=C18—C19 torsion angle being 8.2 (2)° (DFT = 0.21°) in (I) and −5.7 (4)° (DFT = −1.06°) in (II). The small deviations between the experimental and DFT values are due to the inter­molecular inter­actions observed in the solid-state environment but absent during the optimization process.

The enone moiety in (I) [O1/C15–C17, maximum deviation of 0.0133 (12) Å at O1] forms dihedral angles of 87.63 (14) and 7.70 (15)°, respectively, with the anthracene ring system [C1–C14, maximum deviation of 0.044 (14) Å at C14] and the nitro­benzene moiety [C18–C23, maximum deviation of 0.007 (14) Å at C18]. Meanwhile in (II), the enone moiety [O1/C15–C17, maximum deviation of 0.033 (3) Å at O1] forms dihedral angles of 82.5 (3) and 6.8 (3)°, respectively, with the anthracene ring system [C1–C14, maximum deviation of 0.031 (5) Å at C4] and the iodo­benzene ring [C18–C23, maximum deviation of 0.002 (3) Å at C18]. The anthracene ring system forms dihedral angles of 87.50 (6)° with the nitro­benzene ring in (I) and of 80.45 (11)° with the iodo­benzene ring in (II). These large dihedral angles may indicate the diminishing electronic effect between the anthracene groups through the enone bridge (Jung *et al.*, 2008 ▸).

## Supra­molecular features   

**Figure 2:**
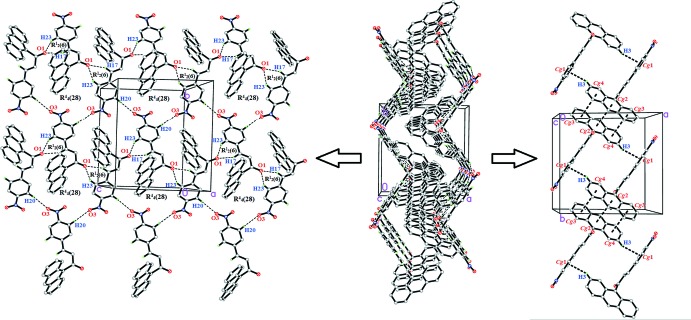
Packing diagram showing weak C—H⋯O, C—H⋯π and π–π inter­actions in (I).

**Figure 3:**
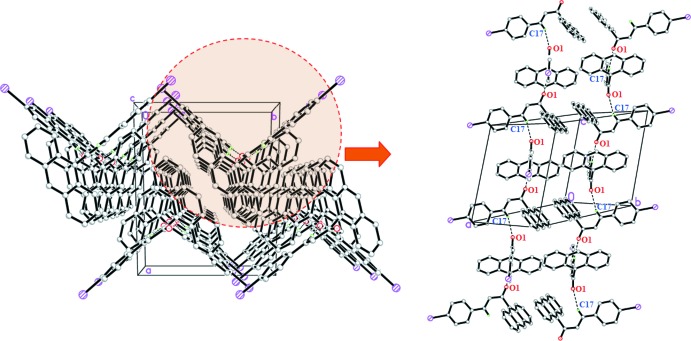
The crystal packing of compound (II) showing weak C—H⋯O inter­actions.

In the crystal of (I), C17—H17*A*⋯O1, C20—H20*A*⋯O3 and C23—H23*B*⋯O1 hydrogen bonds link the mol­ecules into dimers, generating 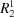
(6) and 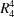
(28) ring motifs (Fig. 2 ▸ and Table 1 ▸). C—H⋯π and π–π inter­actions [*Cg*2⋯*Cg*2(1 − *x*, −*y*, 1 − *z*) = 3.6900 (9) Å and *Cg*3⋯*Cg*4(1 − *x*, −*y*, 1 − *z*) = 3.7214 (10) Å; *Cg*1, *Cg*2, *Cg*3, *Cg*4 are the centroids of the C18–C23, C1/C6–C8/C13/C14, C8–C13 and C1–C6 rings, respectively] further stabilize the crystal structure, forming a three-dimensional network. In the crystal of (II), C—H⋯O hydrogen bonds (Table 2 ▸) link the mol­ecules into infinite chains along the *c*-axis direction (Fig. 3 ▸).

## UV–Vis absorption analysis and frontier mol­ecular orbital (FMO) energies   

**Figure 4:**
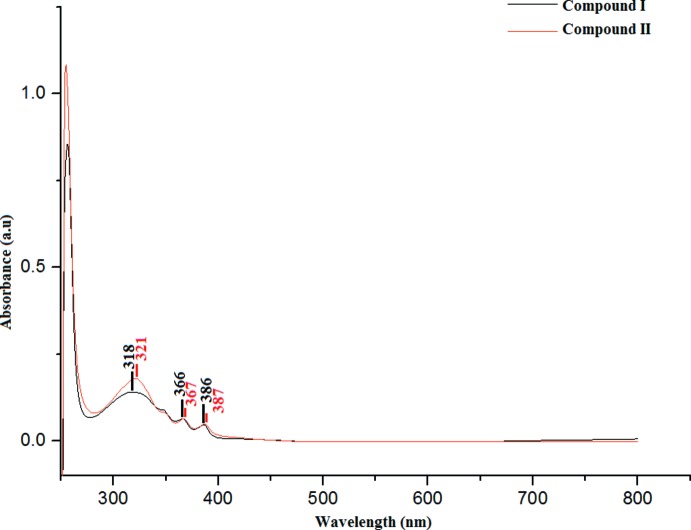
UV–Vis absorption spectra for compounds (I) and (II).

TD–DFT calculations at the B3LYP/6-311G++(d,p) level were performed to simulate the absorption characteristics and obtain information about the excited states. The experimental spectrum (Fig. 4 ▸) shows peaks at wavelengths of 318, 366 and 386 nm in (I) and 321, 367 and 387 nm in (II) with the wavelength of maximum absorbance being observed at 386 nm in (I) and 387 nm in (II). The absorption maxima are assigned to the π–π^*^ transitions, *i.e*. the transition of an electron from a bonding (π) to an anti-bonding (π^*^) mol­ecular orbital, which are attributed to the C=O groups and aromatic ring excitations. The experimentally measured spectra of both compounds match those of the simulated chalcones, which have maxima at 395 nm for (I) and 394 nm for (II).

**Figure 5:**
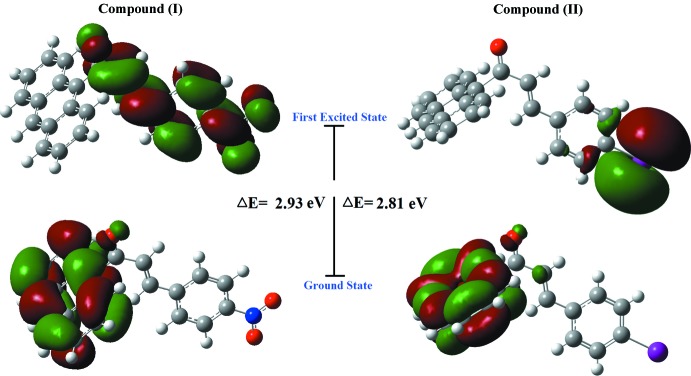
The electron distribution of the HOMO and LUMO energy levels in compounds (I) and (II).

The difference in energy of the HOMO and LUMO is an important index that provides information about the chemical stability of mol­ecules since these energies are directly related to the ability to donate and accept electrons. In the ground state (HOMO), the charge densities are mainly delocalized over the anthracene ring systems and the enone moiety, while in the LUMO state, the charge densities are accumulated on the nitro­benzene ring and the enone moiety in (I), and the iodo­benzene ring in (II). A small HOMO–LUMO gap automatically means small excitation energies to the manifold excited states and a large HOMO–LUMO gap implies high stability with respect to chemical reactions (Custodio *et al.*, 2017 ▸). The HOMO–LUMO energy gaps (Fig. 5 ▸) are computed to be 2.93 eV and 2.81 eV, respectively, for (I) and (II). In the experimental results, the value of energy gap was estimated from the absorption curve by extrapolating the linear portion of the curve to zero absorption, giving values of 3.14 eV for (I) and 3.07 eV for (II). These values for the band gaps suggest that the materials are dielectric in nature (Suguna *et al.*, 2015 ▸), dielectric materials having wide transparency in the UV region. Such materials with wide transparency are required for the fabrication of optical electronic devices.

## Mol­ecular electrostatic potential (MEP)   

**Figure 6:**
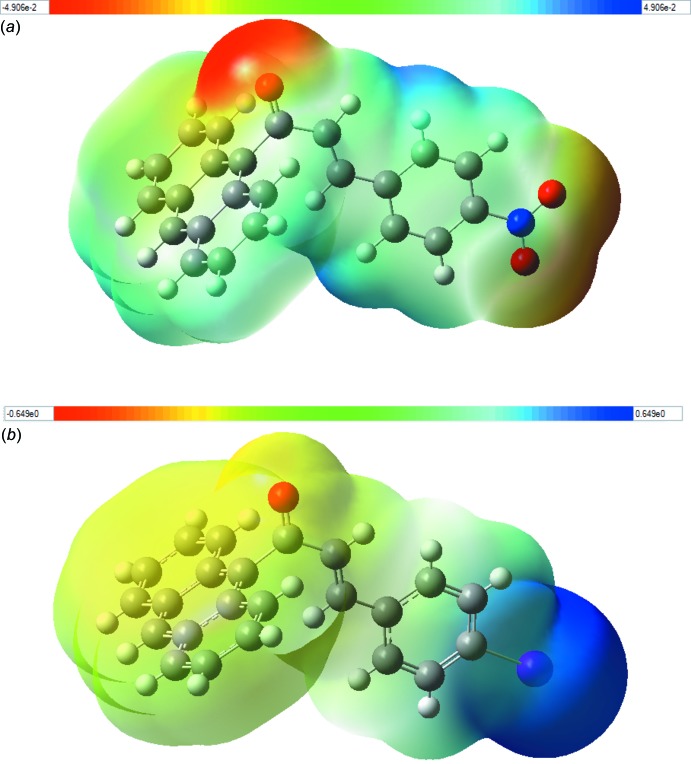
Three-dimensional maps of the total electron density surface of (*a*) compound (I) and (*b*) compound (II) with electrostatic potential calculated at B3LYP/6–311 G++ (d,p) level.

The importance of the MEP lies in the fact that it simultaneously displays mol­ecular size and shape as well as positive, negative and neutral electrostatic potential regions in terms of colour grading and is useful in investigating relationships between mol­ecular structure and physicochemical properties (Murray & Sen, 1996 ▸; Scrocco & Tomasi, 1978 ▸). The MEP maps for the mol­ecules of (I) and (II) were calculated theoretically at the B3LYP/6-311G++(d,p) level of theory and the obtained plots are shown in Fig. 6 ▸. The negative red regions are concentrated at the oxygen atoms, showing the electrophilic sites. Hence, the oxygen atoms are the most reactive sites for nucleophilic attack, as well as the more proper sites to attack the positive regions of the receptor mol­ecule. The negative potential values of compounds (I) and (II) are −0.049 a.u and −0.649 a.u., respectively. The blue regions indicate areas of positive charge concentration, which are concentrated over the hydrogen atoms and iodine substituent atom, indicating the nucleophilic sites. Green regions represent areas with zero potential.

## Database survey   

A survey of the Cambridge Structural Database (CSD, Version 5.39, last update November 2017; Groom *et al.*, 2016 ▸) revealed fused-ring substituted chalcones similar to the title compounds. There are four compounds that have an anthrancene-ketone substituent on the chalcone, *viz*. 9-anthryl styryl ketone and 9,10-anthryl bis­(styryl ketone) (Harlow *et al.*, 1975 ▸), (2*E*)-1-(anthracen-9-yl)-3-[4-(propan-2-yl)phen­yl]prop-2-en-1-one (Girisha *et al.*, 2016 ▸), and (*E*)-1-(anthracen-9-yl)-3-(2-chloro-6-fluoro­phen­yl)prop-2-en-1-one (Abdullah *et al.*, 2016 ▸). Zainuri *et al.* (2018*c* ▸) reported the structure of (*E*)-1,3-bis­(anthracen-9-yl)prop-2-en-1-one. Others related compounds include 1-(anthracen-9-yl)-2-meth­ylprop-2-en-1-one (Agrahari *et al.*, 2015 ▸) and 9-anthroylacetone (Cicogna *et al.*, 2004 ▸).

## Synthesis and crystallization   

9-Acetyl­anthrancene (0.5 mmol) was dissolved in methanol (20 ml) for about 10–15 mins. Then 4-nitro­benzaldehyde (0.5 mmol) [for (I)] or 4-iodo­benzaldehye (0.5 mmol) [for (II)] was added and the solution was stirred for another 10–15 min. Then, NaOH was added and after stirring for 5 h, the reaction mixture was poured into cold water (50 ml) and stirred for 5–10 min. The precipitated solid was filtered, dried and recrystallized from acetone solution to obtain the corresponding chalcones.

## Refinement   

Crystal data collection and structure refinement details are summarized in Table 3 ▸. All H atoms were positioned geometrically [C—H = 0.95 Å in (I) and 0.93 Å in (II)] and refined using a riding model with *U*_iso_(H) = 1.2*U*_eq_(C). In the final refinement of compound (II), three outliers (316, 232, 114) were omitted.

## Supplementary Material

Crystal structure: contains datablock(s) I, II, global. DOI: 10.1107/S2056989018012641/xu5939sup1.cif

Structure factors: contains datablock(s) I. DOI: 10.1107/S2056989018012641/xu5939Isup5.hkl

Structure factors: contains datablock(s) II. DOI: 10.1107/S2056989018012641/xu5939IIsup6.hkl

Supporting information file. DOI: 10.1107/S2056989018012641/xu5939Isup4.cml

Supporting information file. DOI: 10.1107/S2056989018012641/xu5939IIsup5.cml

CCDC references: 1817222, 1817220

Additional supporting information:  crystallographic information; 3D view; checkCIF report

## Figures and Tables

**Table 1:** Hydrogen-bond geometry (Å, °) for (I) *Cg*1 is the centroid of the C18–C23 ring.

*D*—H⋯*A*	*D*—H	H⋯*A*	*D*⋯*A*	*D*—H⋯*A*
C17—H17*A*⋯O1^i^	0.95	2.35	3.2279 (18)	154
C20—H20*A*⋯O3^ii^	0.95	2.45	3.336 (2)	156
C23—H23*B*⋯O1^i^	0.95	2.53	3.3763 (18)	148
C3—H3*A*⋯*Cg*1^iii^	0.95	2.78	3.6179 (19)	148

Symmetry codes: (i) 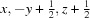; (ii) 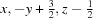; (iii) 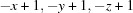.

**Table 2:** Hydrogen-bond geometry (Å, °) for (II)

*D*—H⋯*A*	*D*—H	H⋯*A*	*D*⋯*A*	*D*—H⋯*A*
C17—H17*A*⋯O1^i^	0.93	2.49	3.369 (3)	158

Symmetry code: (i) 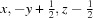.

**Table 3:** Experimental details

	(I)	(II)
Crystal data		
Chemical formula	C_23_H_15_NO_3_	C_23_H_15_IO
*M*_r_	353.36	434.25
Crystal system, space group	Monoclinic, *P*2_1_/*c*	Monoclinic, *P*2_1_/*c*
Temperature (K)	100	294
*a*, *b*, *c* (Å)	12.9197 (14), 12.7282 (13), 10.9016 (12)	14.8004 (12), 11.3095 (9), 11.5139 (9)
β (°)	105.212 (2)	111.3608 (13)
*V* (Å^3^)	1729.9 (3)	1794.9 (2)
*Z*	4	4
Radiation type	Mo *K*α	Mo *K*α
μ (mm^−1^)	0.09	1.79
Crystal size (mm)	0.51 × 0.23 × 0.12	0.24 × 0.20 × 0.20

Data collection		
Diffractometer	Bruker SMART APEXII DUO CCD area detector	Bruker SMART APEXII DUO CCD area detector
Absorption correction	Multi-scan (*SADABS*; Bruker, 2009 ▸)	Multi-scan (*SADABS*; Bruker, 2009 ▸)
No. of measured, independent and observed [*I* > 2σ(*I*)] reflections	36707, 4862, 3416	20023, 5265, 3933
*R*_int_	0.076	0.027
(sin θ/λ)_max_ (Å^−1^)	0.695	0.706

Refinement		
*R*[*F* ^2^ > 2σ(*F* ^2^)], *wR*(*F* ^2^), *S*	0.051, 0.136, 1.03	0.034, 0.097, 1.02
No. of reflections	4862	5265
No. of parameters	244	226
H-atom treatment	H-atom parameters constrained	H-atom parameters constrained
Δρ_max_, Δρ_min_ (e Å^−3^)	0.32, −0.22	1.24, −1.01

Computer programs: *APEX2* and *SAINT* (Bruker, 2009 ▸), *SHELXL2014* (Sheldrick, 2015 ▸), *SHELXTL* (Sheldrick, 2008 ▸) and *PLATON* (Spek, 2009 ▸).

## supplementary crystallographic information

### (E)-1-(Anthracen-9-yl)-3-(4-nitrophenyl)prop-2-en-1-one (I) Crystal data

**Table:**

C_23_H_15_NO_3_	*F*(000) = 736
*M**_r_* = 353.36	*D*_x_ = 1.357 Mg m^−^^3^
Monoclinic, *P*2_1_/*c*	Mo *K*α radiation, λ = 0.71073 Å
*a* = 12.9197 (14) Å	Cell parameters from 4370 reflections
*b* = 12.7282 (13) Å	θ = 2.3–29.2°
*c* = 10.9016 (12) Å	µ = 0.09 mm^−^^1^
β = 105.212 (2)°	*T* = 100 K
*V* = 1729.9 (3) Å^3^	Block, bronze
*Z* = 4	0.51 × 0.23 × 0.12 mm

### (E)-1-(Anthracen-9-yl)-3-(4-nitrophenyl)prop-2-en-1-one (I) Data collection

**Table:**

Bruker SMART APEXII DUO CCD area-detector diffractometer	3416 reflections with *I* > 2σ(*I*)
Radiation source: fine-focus sealed tube	*R*_int_ = 0.076
φ and ω scans	θ_max_ = 29.6°, θ_min_ = 1.6°
Absorption correction: multi-scan (*SADABS*; Bruker, 2009)	*h* = −17→17
	*k* = −17→17
36707 measured reflections	*l* = −15→15
4862 independent reflections	

### (E)-1-(Anthracen-9-yl)-3-(4-nitrophenyl)prop-2-en-1-one (I) Refinement

**Table:**

Refinement on *F*^2^	0 restraints
Least-squares matrix: full	Hydrogen site location: inferred from neighbouring sites
*R*[*F*^2^ > 2σ(*F*^2^)] = 0.051	H-atom parameters constrained
*wR*(*F*^2^) = 0.136	*w* = 1/[σ^2^(*F*_o_^2^) + (0.0515*P*)^2^ + 0.7203*P*] where *P* = (*F*_o_^2^ + 2*F*_c_^2^)/3
*S* = 1.03	(Δ/σ)_max_ < 0.001
4862 reflections	Δρ_max_ = 0.32 e Å^−^^3^
244 parameters	Δρ_min_ = −0.22 e Å^−^^3^

### (E)-1-(Anthracen-9-yl)-3-(4-nitrophenyl)prop-2-en-1-one (I) Special details

**Table:**

Geometry. All esds (except the esd in the dihedral angle between two l.s. planes) are estimated using the full covariance matrix. The cell esds are taken into account individually in the estimation of esds in distances, angles and torsion angles; correlations between esds in cell parameters are only used when they are defined by crystal symmetry. An approximate (isotropic) treatment of cell esds is used for estimating esds involving l.s. planes.

### (E)-1-(Anthracen-9-yl)-3-(4-nitrophenyl)prop-2-en-1-one (I) Fractional atomic coordinates and isotropic or equivalent isotropic displacement parameters (Å^2^)

**Table:**

	*x*	*y*	*z*	*U*_iso_*/*U*_eq_	
N1	0.00262 (10)	0.72156 (11)	0.47682 (13)	0.0289 (3)	
O1	0.34795 (9)	0.19601 (9)	0.21866 (10)	0.0300 (3)	
O2	−0.04488 (12)	0.76881 (11)	0.38140 (13)	0.0508 (4)	
O3	0.00316 (10)	0.74898 (9)	0.58507 (12)	0.0361 (3)	
C1	0.48427 (11)	0.19570 (11)	0.50953 (13)	0.0199 (3)	
C2	0.54467 (12)	0.27977 (12)	0.47617 (14)	0.0245 (3)	
H2A	0.5135	0.3220	0.4041	0.029*	
C3	0.64652 (13)	0.30025 (13)	0.54624 (15)	0.0288 (3)	
H3A	0.6852	0.3569	0.5229	0.035*	
C4	0.69550 (13)	0.23788 (13)	0.65380 (15)	0.0288 (3)	
H4A	0.7668	0.2527	0.7015	0.035*	
C5	0.64081 (12)	0.15728 (13)	0.68864 (14)	0.0254 (3)	
H5A	0.6744	0.1160	0.7607	0.030*	
C6	0.53356 (11)	0.13328 (11)	0.61891 (13)	0.0210 (3)	
C7	0.47693 (12)	0.04993 (11)	0.65304 (13)	0.0230 (3)	
H7A	0.5107	0.0074	0.7238	0.028*	
C8	0.37195 (12)	0.02766 (11)	0.58571 (13)	0.0221 (3)	
C9	0.31336 (13)	−0.05676 (12)	0.62076 (15)	0.0274 (3)	
H9A	0.3468	−0.0999	0.6911	0.033*	
C10	0.21033 (14)	−0.07675 (13)	0.55522 (16)	0.0316 (4)	
H10A	0.1728	−0.1338	0.5797	0.038*	
C11	0.15852 (13)	−0.01261 (13)	0.45023 (15)	0.0298 (4)	
H11A	0.0860	−0.0261	0.4062	0.036*	
C12	0.21207 (12)	0.06794 (12)	0.41231 (14)	0.0248 (3)	
H12A	0.1766	0.1097	0.3415	0.030*	
C13	0.32097 (11)	0.09053 (11)	0.47751 (13)	0.0211 (3)	
C14	0.37944 (11)	0.17191 (11)	0.44012 (12)	0.0190 (3)	
C15	0.33164 (11)	0.22957 (11)	0.31720 (13)	0.0213 (3)	
C16	0.26653 (11)	0.32348 (11)	0.31726 (13)	0.0218 (3)	
H16A	0.2392	0.3604	0.2398	0.026*	
C17	0.24430 (11)	0.35893 (11)	0.42279 (13)	0.0216 (3)	
H17A	0.2735	0.3206	0.4988	0.026*	
C18	0.17937 (11)	0.45104 (11)	0.43267 (13)	0.0206 (3)	
C19	0.12077 (12)	0.50663 (12)	0.32612 (14)	0.0231 (3)	
H19A	0.1205	0.4830	0.2434	0.028*	
C20	0.06347 (11)	0.59521 (12)	0.33999 (14)	0.0227 (3)	
H20A	0.0246	0.6333	0.2676	0.027*	
C21	0.06365 (11)	0.62771 (11)	0.46145 (14)	0.0224 (3)	
C22	0.11872 (11)	0.57411 (12)	0.56955 (14)	0.0231 (3)	
H22A	0.1169	0.5971	0.6519	0.028*	
C23	0.17660 (11)	0.48568 (11)	0.55348 (13)	0.0219 (3)	
H23B	0.2152	0.4478	0.6262	0.026*	

### (E)-1-(Anthracen-9-yl)-3-(4-nitrophenyl)prop-2-en-1-one (I) Atomic displacement parameters (Å^2^)

**Table:**

	*U*^11^	*U*^22^	*U*^33^	*U*^12^	*U*^13^	*U*^23^
N1	0.0239 (6)	0.0261 (7)	0.0346 (7)	0.0008 (5)	0.0039 (5)	−0.0038 (6)
O1	0.0371 (6)	0.0323 (6)	0.0197 (5)	0.0029 (5)	0.0058 (5)	−0.0037 (4)
O2	0.0567 (9)	0.0465 (8)	0.0415 (8)	0.0285 (7)	−0.0007 (6)	0.0012 (6)
O3	0.0371 (7)	0.0314 (6)	0.0393 (7)	0.0031 (5)	0.0091 (5)	−0.0098 (5)
C1	0.0212 (7)	0.0207 (7)	0.0181 (6)	0.0018 (5)	0.0057 (5)	−0.0020 (5)
C2	0.0256 (7)	0.0262 (7)	0.0215 (7)	−0.0016 (6)	0.0058 (6)	0.0005 (6)
C3	0.0261 (8)	0.0314 (8)	0.0290 (8)	−0.0067 (6)	0.0076 (6)	−0.0013 (6)
C4	0.0219 (7)	0.0364 (9)	0.0262 (8)	−0.0004 (6)	0.0030 (6)	−0.0051 (6)
C5	0.0230 (7)	0.0318 (8)	0.0195 (7)	0.0049 (6)	0.0020 (6)	−0.0003 (6)
C6	0.0224 (7)	0.0214 (7)	0.0193 (6)	0.0036 (6)	0.0054 (5)	−0.0023 (5)
C7	0.0280 (8)	0.0219 (7)	0.0188 (6)	0.0050 (6)	0.0056 (6)	0.0014 (5)
C8	0.0273 (7)	0.0195 (7)	0.0204 (7)	0.0025 (6)	0.0082 (6)	−0.0028 (5)
C9	0.0381 (9)	0.0223 (7)	0.0238 (7)	−0.0002 (6)	0.0116 (6)	0.0004 (6)
C10	0.0408 (9)	0.0252 (8)	0.0328 (8)	−0.0094 (7)	0.0165 (7)	−0.0029 (7)
C11	0.0281 (8)	0.0312 (8)	0.0310 (8)	−0.0069 (7)	0.0090 (6)	−0.0065 (7)
C12	0.0252 (7)	0.0257 (7)	0.0230 (7)	0.0003 (6)	0.0053 (6)	−0.0021 (6)
C13	0.0231 (7)	0.0209 (7)	0.0202 (7)	0.0002 (6)	0.0074 (5)	−0.0028 (5)
C14	0.0225 (7)	0.0177 (6)	0.0168 (6)	0.0028 (5)	0.0051 (5)	−0.0023 (5)
C15	0.0203 (7)	0.0228 (7)	0.0195 (7)	−0.0038 (6)	0.0029 (5)	−0.0004 (5)
C16	0.0232 (7)	0.0217 (7)	0.0188 (6)	−0.0006 (6)	0.0025 (5)	0.0023 (5)
C17	0.0217 (7)	0.0214 (7)	0.0196 (6)	−0.0011 (5)	0.0017 (5)	0.0019 (5)
C18	0.0202 (7)	0.0200 (7)	0.0215 (7)	−0.0021 (5)	0.0051 (5)	0.0010 (5)
C19	0.0230 (7)	0.0254 (7)	0.0205 (7)	−0.0022 (6)	0.0049 (6)	0.0007 (6)
C20	0.0204 (7)	0.0235 (7)	0.0224 (7)	−0.0001 (6)	0.0025 (5)	0.0039 (6)
C21	0.0184 (7)	0.0191 (7)	0.0292 (7)	−0.0022 (5)	0.0053 (6)	−0.0012 (6)
C22	0.0215 (7)	0.0241 (7)	0.0233 (7)	−0.0054 (6)	0.0050 (6)	−0.0027 (6)
C23	0.0220 (7)	0.0217 (7)	0.0201 (7)	−0.0021 (6)	0.0022 (5)	0.0019 (5)

### (E)-1-(Anthracen-9-yl)-3-(4-nitrophenyl)prop-2-en-1-one (I) Geometric parameters (Å, º)

**Table:**

N1—O2	1.2187 (18)	C10—H10A	0.9500
N1—O3	1.2289 (17)	C11—C12	1.361 (2)
N1—C21	1.4650 (19)	C11—H11A	0.9500
O1—C15	1.2246 (17)	C12—C13	1.429 (2)
C1—C14	1.4002 (19)	C12—H12A	0.9500
C1—C2	1.427 (2)	C13—C14	1.404 (2)
C1—C6	1.4354 (19)	C14—C15	1.5104 (19)
C2—C3	1.362 (2)	C15—C16	1.462 (2)
C2—H2A	0.9500	C16—C17	1.335 (2)
C3—C4	1.420 (2)	C16—H16A	0.9500
C3—H3A	0.9500	C17—C18	1.462 (2)
C4—C5	1.355 (2)	C17—H17A	0.9500
C4—H4A	0.9500	C18—C23	1.398 (2)
C5—C6	1.428 (2)	C18—C19	1.4006 (19)
C5—H5A	0.9500	C19—C20	1.379 (2)
C6—C7	1.394 (2)	C19—H19A	0.9500
C7—C8	1.392 (2)	C20—C21	1.387 (2)
C7—H7A	0.9500	C20—H20A	0.9500
C8—C9	1.423 (2)	C21—C22	1.386 (2)
C8—C13	1.435 (2)	C22—C23	1.388 (2)
C9—C10	1.359 (2)	C22—H22A	0.9500
C9—H9A	0.9500	C23—H23B	0.9500
C10—C11	1.422 (2)		
			
O2—N1—O3	123.65 (14)	C11—C12—C13	120.88 (14)
O2—N1—C21	118.06 (13)	C11—C12—H12A	119.6
O3—N1—C21	118.28 (13)	C13—C12—H12A	119.6
C14—C1—C2	122.73 (13)	C14—C13—C12	122.58 (13)
C14—C1—C6	118.98 (13)	C14—C13—C8	118.97 (13)
C2—C1—C6	118.28 (13)	C12—C13—C8	118.45 (13)
C3—C2—C1	120.88 (14)	C1—C14—C13	121.37 (13)
C3—C2—H2A	119.6	C1—C14—C15	119.12 (12)
C1—C2—H2A	119.6	C13—C14—C15	119.35 (12)
C2—C3—C4	120.78 (15)	O1—C15—C16	121.13 (13)
C2—C3—H3A	119.6	O1—C15—C14	118.98 (13)
C4—C3—H3A	119.6	C16—C15—C14	119.89 (12)
C5—C4—C3	120.16 (14)	C17—C16—C15	122.05 (13)
C5—C4—H4A	119.9	C17—C16—H16A	119.0
C3—C4—H4A	119.9	C15—C16—H16A	119.0
C4—C5—C6	121.15 (14)	C16—C17—C18	126.28 (13)
C4—C5—H5A	119.4	C16—C17—H17A	116.9
C6—C5—H5A	119.4	C18—C17—H17A	116.9
C7—C6—C5	121.66 (13)	C23—C18—C19	118.68 (13)
C7—C6—C1	119.58 (13)	C23—C18—C17	118.64 (13)
C5—C6—C1	118.75 (13)	C19—C18—C17	122.67 (13)
C8—C7—C6	121.44 (13)	C20—C19—C18	120.74 (14)
C8—C7—H7A	119.3	C20—C19—H19A	119.6
C6—C7—H7A	119.3	C18—C19—H19A	119.6
C7—C8—C9	121.72 (14)	C19—C20—C21	118.83 (13)
C7—C8—C13	119.59 (13)	C19—C20—H20A	120.6
C9—C8—C13	118.70 (14)	C21—C20—H20A	120.6
C10—C9—C8	121.11 (15)	C22—C21—C20	122.48 (14)
C10—C9—H9A	119.4	C22—C21—N1	118.42 (13)
C8—C9—H9A	119.4	C20—C21—N1	119.10 (13)
C9—C10—C11	120.31 (15)	C21—C22—C23	117.73 (13)
C9—C10—H10A	119.8	C21—C22—H22A	121.1
C11—C10—H10A	119.8	C23—C22—H22A	121.1
C12—C11—C10	120.51 (15)	C22—C23—C18	121.51 (13)
C12—C11—H11A	119.7	C22—C23—H23B	119.2
C10—C11—H11A	119.7	C18—C23—H23B	119.2
			
C14—C1—C2—C3	−179.96 (14)	C6—C1—C14—C15	−173.44 (12)
C6—C1—C2—C3	0.0 (2)	C12—C13—C14—C1	176.75 (13)
C1—C2—C3—C4	0.5 (2)	C8—C13—C14—C1	−2.9 (2)
C2—C3—C4—C5	−0.5 (2)	C12—C13—C14—C15	−8.0 (2)
C3—C4—C5—C6	−0.1 (2)	C8—C13—C14—C15	172.35 (12)
C4—C5—C6—C7	179.32 (14)	C1—C14—C15—O1	86.18 (17)
C4—C5—C6—C1	0.6 (2)	C13—C14—C15—O1	−89.21 (17)
C14—C1—C6—C7	0.7 (2)	C1—C14—C15—C16	−94.21 (16)
C2—C1—C6—C7	−179.32 (13)	C13—C14—C15—C16	90.40 (16)
C14—C1—C6—C5	179.43 (13)	O1—C15—C16—C17	177.30 (14)
C2—C1—C6—C5	−0.6 (2)	C14—C15—C16—C17	−2.3 (2)
C5—C6—C7—C8	179.20 (14)	C15—C16—C17—C18	−179.35 (13)
C1—C6—C7—C8	−2.1 (2)	C16—C17—C18—C23	−171.08 (14)
C6—C7—C8—C9	−179.44 (14)	C16—C17—C18—C19	8.2 (2)
C6—C7—C8—C13	1.0 (2)	C23—C18—C19—C20	1.5 (2)
C7—C8—C9—C10	179.07 (15)	C17—C18—C19—C20	−177.79 (13)
C13—C8—C9—C10	−1.3 (2)	C18—C19—C20—C21	−0.8 (2)
C8—C9—C10—C11	−0.5 (2)	C19—C20—C21—C22	−0.4 (2)
C9—C10—C11—C12	1.5 (2)	C19—C20—C21—N1	−179.78 (13)
C10—C11—C12—C13	−0.6 (2)	O2—N1—C21—C22	179.39 (15)
C11—C12—C13—C14	179.01 (14)	O3—N1—C21—C22	0.2 (2)
C11—C12—C13—C8	−1.3 (2)	O2—N1—C21—C20	−1.2 (2)
C7—C8—C13—C14	1.5 (2)	O3—N1—C21—C20	179.62 (14)
C9—C8—C13—C14	−178.08 (13)	C20—C21—C22—C23	1.0 (2)
C7—C8—C13—C12	−178.19 (13)	N1—C21—C22—C23	−179.68 (13)
C9—C8—C13—C12	2.2 (2)	C21—C22—C23—C18	−0.3 (2)
C2—C1—C14—C13	−178.15 (13)	C19—C18—C23—C22	−1.0 (2)
C6—C1—C14—C13	1.9 (2)	C17—C18—C23—C22	178.37 (13)
C2—C1—C14—C15	6.6 (2)		

### (E)-1-(Anthracen-9-yl)-3-(4-nitrophenyl)prop-2-en-1-one (I) Hydrogen-bond geometry (Å, º)

Cg1 is the centroid of the C18–C23 ring.

**Table:**

*D*—H···*A*	*D*—H	H···*A*	*D*···*A*	*D*—H···*A*
C17—H17*A*···O1^i^	0.95	2.35	3.2279 (18)	154
C20—H20*A*···O3^ii^	0.95	2.45	3.336 (2)	156
C23—H23*B*···O1^i^	0.95	2.53	3.3763 (18)	148
C3—H3*A*···*Cg*1^iii^	0.95	2.78	3.6179 (19)	148

Symmetry codes: (i) *x*, −*y*+1/2, *z*+1/2; (ii) *x*, −*y*+3/2, *z*−1/2; (iii) −*x*+1, −*y*+1, −*z*+1.

### (E)-1-(Anthracen-9-yl)-3-(4-iodophenyl)prop-2-en-1-one (II) Crystal data

**Table:**

C_23_H_15_IO	*F*(000) = 856
*M**_r_* = 434.25	*D*_x_ = 1.607 Mg m^−^^3^
Monoclinic, *P*2_1_/*c*	Mo *K*α radiation, λ = 0.71073 Å
*a* = 14.8004 (12) Å	Cell parameters from 6699 reflections
*b* = 11.3095 (9) Å	θ = 2.3–27.3°
*c* = 11.5139 (9) Å	µ = 1.79 mm^−^^1^
β = 111.3608 (13)°	*T* = 294 K
*V* = 1794.9 (2) Å^3^	Block, colourless
*Z* = 4	0.24 × 0.20 × 0.20 mm

### (E)-1-(Anthracen-9-yl)-3-(4-iodophenyl)prop-2-en-1-one (II) Data collection

**Table:**

Bruker SMART APEXII DUO CCD area-detector diffractometer	3933 reflections with *I* > 2σ(*I*)
Radiation source: fine-focus sealed tube	*R*_int_ = 0.027
φ and ω scans	θ_max_ = 30.1°, θ_min_ = 1.5°
Absorption correction: multi-scan (*SADABS*; Bruker, 2009)	*h* = −20→20
	*k* = −15→14
20023 measured reflections	*l* = −16→14
5265 independent reflections	

### (E)-1-(Anthracen-9-yl)-3-(4-iodophenyl)prop-2-en-1-one (II) Refinement

**Table:**

Refinement on *F*^2^	0 restraints
Least-squares matrix: full	Hydrogen site location: inferred from neighbouring sites
*R*[*F*^2^ > 2σ(*F*^2^)] = 0.034	H-atom parameters constrained
*wR*(*F*^2^) = 0.097	*w* = 1/[σ^2^(*F*_o_^2^) + (0.0437*P*)^2^ + 1.0689*P*] where *P* = (*F*_o_^2^ + 2*F*_c_^2^)/3
*S* = 1.02	(Δ/σ)_max_ = 0.001
5265 reflections	Δρ_max_ = 1.24 e Å^−^^3^
226 parameters	Δρ_min_ = −1.00 e Å^−^^3^

### (E)-1-(Anthracen-9-yl)-3-(4-iodophenyl)prop-2-en-1-one (II) Special details

**Table:**

Geometry. All esds (except the esd in the dihedral angle between two l.s. planes) are estimated using the full covariance matrix. The cell esds are taken into account individually in the estimation of esds in distances, angles and torsion angles; correlations between esds in cell parameters are only used when they are defined by crystal symmetry. An approximate (isotropic) treatment of cell esds is used for estimating esds involving l.s. planes.

### (E)-1-(Anthracen-9-yl)-3-(4-iodophenyl)prop-2-en-1-one (II) Fractional atomic coordinates and isotropic or equivalent isotropic displacement parameters (Å^2^)

**Table:**

	*x*	*y*	*z*	*U*_iso_*/*U*_eq_	
I1	1.03943 (2)	−0.28858 (2)	0.01034 (2)	0.05842 (9)	
O1	0.68177 (19)	0.2750 (2)	0.2834 (2)	0.0690 (6)	
C1	0.53340 (19)	0.2882 (2)	0.0026 (2)	0.0475 (6)	
C2	0.4848 (3)	0.1961 (3)	0.0395 (4)	0.0655 (8)	
H2A	0.5192	0.1489	0.1073	0.079*	
C3	0.3886 (3)	0.1757 (4)	−0.0231 (4)	0.0838 (12)	
H3A	0.3576	0.1151	0.0024	0.101*	
C4	0.3360 (3)	0.2463 (5)	−0.1264 (4)	0.0885 (13)	
H4A	0.2703	0.2319	−0.1682	0.106*	
C5	0.3790 (2)	0.3341 (4)	−0.1658 (3)	0.0730 (10)	
H5A	0.3426	0.3797	−0.2341	0.088*	
C6	0.4803 (2)	0.3584 (3)	−0.1039 (2)	0.0533 (7)	
C7	0.5266 (2)	0.4483 (3)	−0.1403 (3)	0.0582 (7)	
H7A	0.4912	0.4942	−0.2089	0.070*	
C8	0.6245 (2)	0.4731 (2)	−0.0782 (3)	0.0505 (6)	
C9	0.6722 (3)	0.5656 (3)	−0.1165 (3)	0.0675 (9)	
H9A	0.6377	0.6104	−0.1863	0.081*	
C10	0.7668 (3)	0.5895 (3)	−0.0534 (4)	0.0730 (10)	
H10A	0.7968	0.6499	−0.0806	0.088*	
C11	0.8204 (2)	0.5236 (3)	0.0532 (3)	0.0653 (8)	
H11A	0.8854	0.5415	0.0964	0.078*	
C12	0.7780 (2)	0.4344 (3)	0.0935 (3)	0.0526 (6)	
H12A	0.8143	0.3919	0.1643	0.063*	
C13	0.67851 (19)	0.4049 (2)	0.0287 (2)	0.0439 (5)	
C14	0.63246 (19)	0.3133 (2)	0.0679 (2)	0.0424 (5)	
C15	0.6865 (2)	0.2445 (3)	0.1837 (2)	0.0473 (6)	
C16	0.7450 (2)	0.1432 (3)	0.1768 (2)	0.0502 (6)	
H16A	0.7728	0.0975	0.2481	0.060*	
C17	0.76109 (19)	0.1121 (2)	0.0741 (2)	0.0457 (5)	
H17A	0.7285	0.1554	0.0023	0.055*	
C18	0.82431 (18)	0.0175 (2)	0.0624 (2)	0.0444 (5)	
C19	0.8844 (2)	−0.0486 (3)	0.1638 (2)	0.0561 (7)	
H19A	0.8841	−0.0334	0.2430	0.067*	
C20	0.9439 (2)	−0.1355 (3)	0.1484 (2)	0.0569 (7)	
H20A	0.9831	−0.1789	0.2168	0.068*	
C21	0.94538 (19)	−0.1584 (2)	0.0315 (2)	0.0465 (5)	
C22	0.8867 (2)	−0.0951 (3)	−0.0709 (2)	0.0513 (6)	
H22A	0.8875	−0.1109	−0.1498	0.062*	
C23	0.82706 (19)	−0.0083 (2)	−0.0546 (2)	0.0489 (6)	
H23A	0.7876	0.0342	−0.1236	0.059*	

### (E)-1-(Anthracen-9-yl)-3-(4-iodophenyl)prop-2-en-1-one (II) Atomic displacement parameters (Å^2^)

**Table:**

	*U*^11^	*U*^22^	*U*^33^	*U*^12^	*U*^13^	*U*^23^
I1	0.05287 (12)	0.06107 (14)	0.06002 (13)	0.00369 (8)	0.01901 (9)	0.00257 (8)
O1	0.0894 (17)	0.0801 (16)	0.0428 (10)	−0.0057 (12)	0.0304 (11)	−0.0093 (10)
C1	0.0471 (14)	0.0522 (15)	0.0459 (13)	−0.0077 (11)	0.0204 (11)	−0.0153 (11)
C2	0.0603 (18)	0.074 (2)	0.0660 (19)	−0.0226 (16)	0.0275 (15)	−0.0128 (16)
C3	0.068 (2)	0.101 (3)	0.090 (3)	−0.037 (2)	0.037 (2)	−0.030 (2)
C4	0.0470 (18)	0.127 (3)	0.088 (3)	−0.018 (2)	0.0204 (19)	−0.051 (3)
C5	0.0531 (17)	0.096 (3)	0.0617 (18)	0.0072 (18)	0.0110 (15)	−0.0286 (19)
C6	0.0494 (14)	0.0646 (18)	0.0452 (13)	0.0073 (13)	0.0163 (11)	−0.0152 (12)
C7	0.0687 (18)	0.0591 (17)	0.0446 (13)	0.0168 (14)	0.0182 (13)	−0.0025 (12)
C8	0.0674 (17)	0.0415 (13)	0.0496 (14)	0.0041 (12)	0.0295 (13)	−0.0039 (11)
C9	0.100 (3)	0.0487 (16)	0.0649 (18)	0.0057 (17)	0.0440 (19)	0.0050 (14)
C10	0.100 (3)	0.0501 (18)	0.089 (2)	−0.0145 (18)	0.058 (2)	−0.0020 (17)
C11	0.0657 (18)	0.0601 (19)	0.083 (2)	−0.0164 (15)	0.0424 (17)	−0.0122 (16)
C12	0.0502 (14)	0.0524 (16)	0.0600 (15)	−0.0081 (12)	0.0256 (13)	−0.0083 (12)
C13	0.0506 (13)	0.0412 (13)	0.0454 (12)	−0.0021 (10)	0.0240 (11)	−0.0072 (10)
C14	0.0457 (13)	0.0436 (13)	0.0407 (12)	−0.0047 (10)	0.0191 (10)	−0.0079 (9)
C15	0.0531 (15)	0.0517 (14)	0.0396 (12)	−0.0132 (12)	0.0200 (11)	−0.0062 (11)
C16	0.0557 (15)	0.0527 (15)	0.0376 (12)	−0.0062 (12)	0.0114 (11)	0.0043 (11)
C17	0.0471 (13)	0.0446 (14)	0.0406 (12)	−0.0066 (11)	0.0104 (10)	0.0033 (10)
C18	0.0463 (13)	0.0460 (13)	0.0374 (11)	−0.0068 (11)	0.0111 (10)	0.0013 (10)
C19	0.0637 (17)	0.0644 (18)	0.0376 (12)	0.0065 (14)	0.0152 (12)	0.0054 (12)
C20	0.0579 (16)	0.0666 (18)	0.0395 (12)	0.0079 (14)	0.0099 (11)	0.0105 (12)
C21	0.0433 (13)	0.0457 (14)	0.0470 (13)	−0.0048 (10)	0.0121 (10)	0.0011 (11)
C22	0.0599 (16)	0.0530 (15)	0.0380 (12)	−0.0036 (12)	0.0141 (11)	−0.0001 (11)
C23	0.0549 (14)	0.0501 (15)	0.0373 (11)	−0.0014 (12)	0.0115 (10)	0.0061 (10)

### (E)-1-(Anthracen-9-yl)-3-(4-iodophenyl)prop-2-en-1-one (II) Geometric parameters (Å, º)

**Table:**

I1—C21	2.100 (3)	C11—C12	1.356 (4)
O1—C15	1.226 (3)	C11—H11A	0.9300
C1—C14	1.411 (3)	C12—C13	1.427 (4)
C1—C2	1.416 (4)	C12—H12A	0.9300
C1—C6	1.431 (4)	C13—C14	1.402 (4)
C2—C3	1.361 (5)	C14—C15	1.499 (4)
C2—H2A	0.9300	C15—C16	1.455 (4)
C3—C4	1.409 (7)	C16—C17	1.336 (4)
C3—H3A	0.9300	C16—H16A	0.9300
C4—C5	1.344 (7)	C17—C18	1.460 (4)
C4—H4A	0.9300	C17—H17A	0.9300
C5—C6	1.432 (4)	C18—C23	1.393 (4)
C5—H5A	0.9300	C18—C19	1.398 (4)
C6—C7	1.374 (5)	C19—C20	1.373 (4)
C7—C8	1.391 (4)	C19—H19A	0.9300
C7—H7A	0.9300	C20—C21	1.379 (4)
C8—C9	1.419 (4)	C20—H20A	0.9300
C8—C13	1.426 (4)	C21—C22	1.383 (4)
C9—C10	1.349 (5)	C22—C23	1.379 (4)
C9—H9A	0.9300	C22—H22A	0.9300
C10—C11	1.407 (5)	C23—H23A	0.9300
C10—H10A	0.9300		
			
C14—C1—C2	122.0 (3)	C13—C12—H12A	119.6
C14—C1—C6	119.0 (2)	C14—C13—C8	119.5 (2)
C2—C1—C6	119.0 (3)	C14—C13—C12	122.3 (3)
C3—C2—C1	120.9 (4)	C8—C13—C12	118.2 (2)
C3—C2—H2A	119.5	C13—C14—C1	120.6 (2)
C1—C2—H2A	119.5	C13—C14—C15	120.3 (2)
C2—C3—C4	120.0 (4)	C1—C14—C15	119.0 (2)
C2—C3—H3A	120.0	O1—C15—C16	120.7 (3)
C4—C3—H3A	120.0	O1—C15—C14	119.5 (3)
C5—C4—C3	121.4 (3)	C16—C15—C14	119.7 (2)
C5—C4—H4A	119.3	C17—C16—C15	123.8 (2)
C3—C4—H4A	119.3	C17—C16—H16A	118.1
C4—C5—C6	120.8 (4)	C15—C16—H16A	118.1
C4—C5—H5A	119.6	C16—C17—C18	127.0 (2)
C6—C5—H5A	119.6	C16—C17—H17A	116.5
C7—C6—C1	119.5 (3)	C18—C17—H17A	116.5
C7—C6—C5	122.5 (3)	C23—C18—C19	117.5 (3)
C1—C6—C5	117.9 (3)	C23—C18—C17	119.2 (2)
C6—C7—C8	122.3 (3)	C19—C18—C17	123.3 (2)
C6—C7—H7A	118.8	C20—C19—C18	121.2 (3)
C8—C7—H7A	118.8	C20—C19—H19A	119.4
C7—C8—C9	122.2 (3)	C18—C19—H19A	119.4
C7—C8—C13	119.1 (3)	C19—C20—C21	119.9 (3)
C9—C8—C13	118.8 (3)	C19—C20—H20A	120.0
C10—C9—C8	121.1 (3)	C21—C20—H20A	120.0
C10—C9—H9A	119.4	C20—C21—C22	120.5 (3)
C8—C9—H9A	119.4	C20—C21—I1	119.5 (2)
C9—C10—C11	120.6 (3)	C22—C21—I1	120.1 (2)
C9—C10—H10A	119.7	C23—C22—C21	119.1 (2)
C11—C10—H10A	119.7	C23—C22—H22A	120.4
C12—C11—C10	120.5 (3)	C21—C22—H22A	120.4
C12—C11—H11A	119.7	C22—C23—C18	121.8 (2)
C10—C11—H11A	119.7	C22—C23—H23A	119.1
C11—C12—C13	120.9 (3)	C18—C23—H23A	119.1
C11—C12—H12A	119.6		
			
C14—C1—C2—C3	178.3 (3)	C12—C13—C14—C1	178.8 (2)
C6—C1—C2—C3	−1.5 (4)	C8—C13—C14—C15	−176.9 (2)
C1—C2—C3—C4	0.3 (6)	C12—C13—C14—C15	1.9 (4)
C2—C3—C4—C5	0.4 (6)	C2—C1—C14—C13	179.8 (3)
C3—C4—C5—C6	0.3 (6)	C6—C1—C14—C13	−0.4 (4)
C14—C1—C6—C7	0.0 (4)	C2—C1—C14—C15	−3.3 (4)
C2—C1—C6—C7	179.8 (3)	C6—C1—C14—C15	176.5 (2)
C14—C1—C6—C5	−177.7 (2)	C13—C14—C15—O1	93.4 (3)
C2—C1—C6—C5	2.1 (4)	C1—C14—C15—O1	−83.5 (3)
C4—C5—C6—C7	−179.1 (3)	C13—C14—C15—C16	−85.8 (3)
C4—C5—C6—C1	−1.5 (5)	C1—C14—C15—C16	97.3 (3)
C1—C6—C7—C8	1.0 (4)	O1—C15—C16—C17	−173.2 (3)
C5—C6—C7—C8	178.6 (3)	C14—C15—C16—C17	6.0 (4)
C6—C7—C8—C9	179.6 (3)	C15—C16—C17—C18	175.2 (2)
C6—C7—C8—C13	−1.4 (4)	C16—C17—C18—C23	175.4 (3)
C7—C8—C9—C10	178.7 (3)	C16—C17—C18—C19	−5.7 (4)
C13—C8—C9—C10	−0.2 (4)	C23—C18—C19—C20	0.0 (4)
C8—C9—C10—C11	−0.6 (5)	C17—C18—C19—C20	−178.9 (3)
C9—C10—C11—C12	0.6 (5)	C18—C19—C20—C21	0.4 (5)
C10—C11—C12—C13	0.3 (5)	C19—C20—C21—C22	−0.6 (4)
C7—C8—C13—C14	1.0 (4)	C19—C20—C21—I1	178.4 (2)
C9—C8—C13—C14	180.0 (2)	C20—C21—C22—C23	0.4 (4)
C7—C8—C13—C12	−177.9 (2)	I1—C21—C22—C23	−178.6 (2)
C9—C8—C13—C12	1.1 (4)	C21—C22—C23—C18	0.1 (4)
C11—C12—C13—C14	−180.0 (3)	C19—C18—C23—C22	−0.2 (4)
C11—C12—C13—C8	−1.1 (4)	C17—C18—C23—C22	178.7 (2)
C8—C13—C14—C1	0.0 (4)		

### (E)-1-(Anthracen-9-yl)-3-(4-iodophenyl)prop-2-en-1-one (II) Hydrogen-bond geometry (Å, º)

**Table:**

*D*—H···*A*	*D*—H	H···*A*	*D*···*A*	*D*—H···*A*
C17—H17*A*···O1^i^	0.93	2.49	3.369 (3)	158

Symmetry code: (i) *x*, −*y*+1/2, *z*−1/2.
